# « Champ-École Paysan », une approche pédagogique participative pour l'amélioration de la lutte contre les vecteurs du paludisme en zone de riziculture irriguée au Bénin

**DOI:** 10.48327/mtsi.v3i3.2023.281

**Published:** 2023-09-24

**Authors:** Innocent DJÈGBÈ, Yêyinou Laura Estelle LOKO, Donald HESSOU-DJOSSOU, Massioudou Koto Yérima GOUNOU BOUKARI, Brice GBAGUIDI, Razack ADÉOTI, Martin AKOGBÉTO, Rousseau DJOUAKA, Fabrice CHANDRE

**Affiliations:** 1 Laboratoire des Sciences naturelles et application, École normale supérieure de Natitingou; Université nationale des sciences, technologies, ingénierie et mathématiques (UNSTIM), BP 72 Natitingou, Bénin; 2 Laboratoire d'Entomologie appliquée (LEnA), École nationale supérieure des biosciences et biotechnologies appliquées (ENSBBA); Université nationale des sciences, technologies, ingénierie et mathématiques (UNSTIM), BP 14, Dassa, Bénin; 3 Plateforme Agriculture-environnement-santé, Institut international d'agriculture tropicale (IITA), 08 BP 0932 Tri postal Cotonou, Bénin; 4 Centre de recherche entomologique de Cotonou (CREC), Ministère de la Santé, 06 BP 2604 Cotonou, Bénin; 5 Maladies infectieuses et vecteurs : Écologie, génétique, évolution et contrôle (MIVEGEC), Montpellier Cedex 5, France

**Keywords:** Paludisme, *Anopheles,*, Riziculture, Labour, Irrigation, Champ-école paysan, Densité larvaire, Malanville, Bénin, Afrique subsaharienne, Malaria, *Anopheles,*, Rice production, Tillage, Flooding, Farmer Field School, Larval density, Malanville, Benin, Sub-Saharan Africa

## Abstract

**Introduction/justification:**

La riziculture irriguée occupe une place importante dans l'économie des pays tropicaux d'Afrique où le paludisme sévit et demeure un problème majeur de santé publique. Concilier l'expansion de la riziculture irriguée et la lutte contre les vecteurs du paludisme s'avère un important défi.

**Matériel et méthodes:**

Afin de vulgariser auprès des producteurs les pratiques agricoles telles que l'irrigation intermittente et le labour minimal, 12 « champs-écoles paysans (CEP) » ont été installés dans le périmètre rizicole de Malanville au nord du Bénin. L'impact de l'irrigation intermittente et du labour minimal dans la réduction des densités larvaires d'anophèles d'une part et de tous les culicidés d'autre part, a été établi par la comparaison des densités des larves au niveau des parcelles tests et témoins.

**Résultats:**

Seuls les systèmes de labour profond et d'irrigation permanente sont actuellement pratiqués dans les rizières de Malanville. À l'issue des CEP, les agriculteurs semblent maîtriser la reconnaissance des gîtes, des larves, et le cycle du développement des moustiques. Le labour minimal combiné à l'irrigation intermittente a réduit significativement la densité des larves de moustiques. Les taux de réduction ont été respectivement de 10,5; 5,4 et 2,5 pendant le repiquage, le tallage et la maturation. Pour les larves du genre *Anopheles,* les taux de réduction ont été respectivement de 16; 5,5 et 4. Le labour minimal combiné à l'irrigation intermittente et le labour profond combiné à l'irrigation intermittente ont des taux de réduction similaires pendant le repiquage et la maturation.

**Discussion/conclusion:**

L'irrigation intermittente associée au labour minimal apparaît efficace pour réduire la densité des larves de moustiques. Elle peut contribuer à la baisse de la nuisance causée par les moustiques et participer à la réduction de l'incidence palustre en milieu rizicole.

## Abstract (en)

**Background & rationale:**

Malaria is a major health problem in Benin where it is the main cause of morbidity and mortality, particularly among children under 5 and pregnant women. Although the vast majority of malaria cases occurs in rural and agricultural areas and are often associated with development projects, very few interventions target the agro-ecosystem. In Benin, irrigated rice growing is expanding to meet the increasing demand of the population. However, continuous flooding and tillage systems induce the development and proliferation of malaria and other diseases vectors. Intermittent flooding of rice plots and minimal tillage can reduce significantly the proliferation of mosquitoes including *Anopheles* in rice fields. However, the dissemination and implementation of these agricultural practices require community-wide action for greater effectiveness. As part of strengthening the capacity of farming communities in the fight against malaria vectors, the “Farmer Field School” appears to be an innovative approach. This learning by doing method promotes interactions between groups of producers to disseminate proven technologies. This study aims to disseminate among rice producers the agricultural practices of intermittent flooding and minimal tillage, likely to reduce the proliferation of malaria vectors in the rice fields.

**Materials & methods:**

This study was carried out in the rice-growing perimeter of Malanville, Benin (11° 52’ 5” North, 3° 22’ 59” East) which covers an area of 516 hectares. Farmer Field Schools were set up after a basic survey at producer level. This survey was carried out through in-depth interview, focus group discussions and direct field observation with producers. Focus groups discussions and interviews made it possible to understand the perception of rice farmers on the link between rice production and the transmission of malaria. In order to disseminate new agricultural practices such as intermittent flooding and minimal tillage among producers, twelve plots have been set up. Farmer Field Schools were monitored weekly with rice producers accompanied by a facilitator and a medical entomologist (learning facilitator or moderator) helping the farmers with the collection and identification of mosquito larvae. According to the different stages of rice development (transplanting, tillering, maturation), the mosquito larvae were collected in the test and control plots from 10 a.m. to 2 p.m. by the dipping method. Then the water in the test compartments (intermittent flooding) was emptied. A cycle of 7 days of flooding and 2 days of drying was carried out for intermittent flooding. Mosquito larvae were identified morphologically using the identification key and *Anopheles* genus larvae were isolated in plastic cups. The impacts of intermittent flooding and minimum tillage in reducing breeding sites and larval densities were established by determining and comparing the larval densities of mosquitoes and of *Anopheles* between the test and control plots.

**Results:**

Direct observations in the field allowed to identify three tillage systems, which include the use of tiller (28%), plow (66%) and hoe (6%) on the rice field. Continuous flooding was the only irrigation system used by farmers. The water used for irrigation comes either from boreholes installed individually or from the Niger River. The volume of water used varies with the seasons, the size of the farms and the variety of rice grown. Farmers observe that the nuisance of mosquitoes increases during the rice production period with an outbreak of malaria cases, especially among children, which leads to crowds in health centers. The preventive measures against malaria among farmers are the use of impregnated mosquito nets distributed free of charge by the national program against malaria, and of insecticide bombs or spirals. Considering the development stages of the rice, the larval densities varied according to the treatments. Overall, minimal tillage applied to intermittent flooding significantly reduced the density of mosquito larvae of all species. The reduction rates were 10.5, 5.4 and 2.5 during transplanting, tillering and maturation, respectively. Considering only the *Anopheles* larvae, minimal tillage applied to intermittent flooding reduced their density by 16, 5.5 and 4 respectively during transplanting, tillering and maturation.

**Discussion/conclusion:**

The rice-growing area of Malanville has many favorable conditions for rice production, including the presence of water supply sources such as the Niger River located near the rice field and numerous boreholes. The availability of water pockets for mosquito breeding during irrigation appeared to contribute to the extension of malaria transmission. The present study showed that the intermittent flooding coupled with minimal tillage could reduce the proliferation of malaria vectors. The results suggested that with technical support to farmers through the “Farmer Field School", the malaria incidence could be reduced in the farming community.

## Introduction

L'Afrique est le continent le plus touché par le paludisme avec 80% des décès qui surviennent en zone subsaharienne [19]. Au Bénin, le paludisme représente 45% des causes de recours aux soins dans les formations sanitaires et se situe au premier rang des principales affections dont souffrent les communautés [22]. Le paludisme étant fortement lié à des espaces partiellement anthropisés [23], les communautés rurales agricoles y sont donc plus exposées [16, 25]. Les agroécosystèmes irrigués tels que les périmètres rizicoles sont des sites potentiels de production de moustiques du genre *Anopheles* [2, 7, 8]. Cela est principalement dû aux systèmes d'irrigation et de labour qui favorisent le développement des gîtes larvaires [14]. Les pratiques agricoles telles que les systèmes traditionnels d'irrigation permanente des casiers rizicoles et les labours profonds dans les champs ont été identifiés comme des facteurs susceptibles de favoriser le développement et la prolifération des moustiques [7, 24].

Au Bénin, la riziculture irriguée est en expansion pour satisfaire la demande croissante des populations [17]. Bien que cet agrosystème augmente les risques de paludisme [3,10,11,20], peu d'interventions de lutte antivectorielle s'y intéressent. Concilier l'expansion de la riziculture irriguée pour l'atteinte de la sécurité alimentaire et la lutte contre les vecteurs du paludisme est un important défi [3].

La lutte contre les vecteurs du paludisme dans un agroécosystème irrigué passe principalement par la gestion de l'eau et par la reconnaissance des anophèles par la population [14]. De nombreuses études ont montré que l'irrigation intermittente des casiers rizicoles et le labour minimal réduisent la production des anophèles dans les rizières [7, 14]. Cependant, la vulgarisation et la mise en œuvre de ces pratiques agricoles nécessitent une action à l'échelle communautaire [23]. Le « champ-école » apparaît comme une approche innovante pouvant faciliter la vulgarisation de ces pratiques. La présente étude vise à vulgariser auprès des producteurs les pratiques agricoles d'irrigation intermittente et du labour minimal susceptibles de réduire la prolifération des vecteurs du paludisme dans les rizières.

## Matériels et méthodes

### Description du milieu d'étude

**Figure 1:**
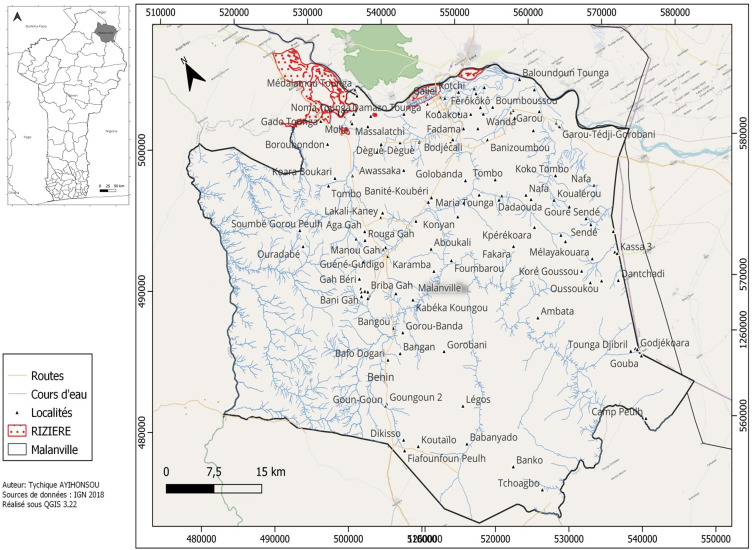
Carte de la commune de Malanville indiquant la zone d'étude (rizières) Map of the city of Malanville showing the study area (rice fields)

La présente étude a été menée dans le périmètre rizicole de Malanville (11° 52’ 5” Nord, 3° 22’ 59” Est) qui couvre une surface de 516 hectares et entoure entièrement la ville (Fig. 1). Le système d'irrigation utilise des forages et l'eau du fleuve Niger. La culture du riz se fait toute l'année avec deux récoltes par an suivant les calendriers de chaque producteur. La transmission du paludisme présente deux pics : un pic lié à la pluviométrie (juin-octobre) et un autre lié à l'irrigation pendant la saison sèche (mars-avril) [7]. Des moustiquaires imprégnées d'insecticide (MII) sont fournies gratuitement aux populations par le Programme national de lutte contre le paludisme [22] avec une priorité accordée aux enfants de moins de 5 ans et aux femmes enceintes. Les enquêtes menées dans cette localité ont montré que l'efficacité des MII est menacée par la résistance aux insecticides des vecteurs du paludisme [6]. Cet ensemble d'informations sur la rizière de Malanville souligne la nécessité d'interagir avec les agriculteurs pour introduire de nouvelles approches afin de réduire la densité des vecteurs du paludisme.

### Mise en place des Champs-Écoles Paysans

#### Collecte d'informations sur les pratiques culturales existantes

Les champs-écoles paysans (CEP) ont été installés après une enquête de base au niveau des producteurs. Cette enquête a été effectuée par des observations directes de terrain et des discussions de groupe incluant des femmes et des hommes producteurs de riz. Afin de permettre à tous les participants d'intervenir dans la discussion, la taille du groupe a été réduite à 8 personnes pour chacun des 12 CEP mis en place. Les observations directes de terrain ont permis de recenser les pratiques agricoles (systèmes de labour, systèmes d'irrigation, sources d'approvisionnement en eau, matériels d'irrigation et de labour). Les discussions de groupe et des interviews ont permis d'appréhender la perception des riziculteurs sur le lien entre la production du riz et la transmission du paludisme.

#### Les essais au champ

Sur les parcelles expérimentales, des casiers rizicoles (5,5 m x 3,0 m; superficie : 16,5 m^2^) ont été cultivés avec divers paramètres : irrigation permanente, irrigation intermittente, labour profond, labour minimal. Deux systèmes de pratiques agricoles, le système d'irrigation permanente et de labour profond (système conventionnel) d'une part et le système d'irrigation intermittente et de labour minimal (système innovateur) d'autre part, ont été comparés:

- Technique 1. Casiers témoins : labour profond et irrigation permanente (LP + IP)
- Technique 2. Casiers tests : labour minimal et irrigation permanente (LM + IP)
- Technique 3. Casiers tests : labour profond et irrigation intermittente (LP + Iin)
- Technique 4. Casiers tests : labour minimal et irrigation intermittente (LM + Iin)

Chaque pratique agricole a été réalisée sur 3 casiers, soit un total de 12 casiers.

### Suivi des essais

Les CEP ont été suivis à une fréquence hebdomadaire par les producteurs de riz, accompagnés d'un facilitateur et d'un entomologiste médical assistant les producteurs dans l'identification et la collecte des larves de moustiques. Suivant les différents stades de développement du riz (repiquage, tallage et maturation), les larves de moustiques ont été collectées dans les casiers tests et témoins, de 10 h à 14 h par la méthode de dipping [21] à l'aide d'une louche d'une capacité de 350 ml (20 prélèvements par casier). Ensuite l'eau des casiers tests (irrigation intermittente) était vidée. Un cycle de 7 jours d'irrigation et de 2 jours de séchage était effectué. Les larves ont été identifiées grâce à la clé d'identification de Gillies et Meillon [9] et celles du genre *Anopheles* isolées dans des gobelets.

### Analyses statistiques

Le nombre de larves de culicidés collectées par louchée, par casier et par séance a été enregistré dans Excel. Les densités ont été comparées par le test de ki2 avec un seuil de significativité fixé à 5%. L'impact de l'irrigation intermittente a été établi par la comparaison des densités des larves de tous les moustiques et la densité des larves d'anophèles entre casiers tests et témoins. Le taux de réduction de la production de larves a été calculé en comparant les casiers témoins et expérimentaux.

## Résultats

### Identification des pratiques culturales dans le périmètre rizicole de Malanville

Les observations directes du terrain ont permis d'identifier trois systèmes de labour : le labour par le motoculteur (28%), le labour à la charrue (66%) et le labour à la bêche *(daba)* (6%). Tous les agriculteurs pratiquent un labour profond et une irrigation permanente. L'eau provient soit des forages installés individuellement sur les parcelles avec emploi de motopompes, soit du fleuve Niger avec un système de pompage et de rigoles permanentes.

En saison sèche, lorsque le niveau d'eau est bas dans le lit du fleuve, ce sont les forages qui sont utilisés. Les parcelles sont habituellement irriguées après le nettoyage du sol afin de faciliter le labour. Après le labour, les casiers sont à nouveau irrigués avant le repiquage.

### Performance des technologies dans la réduction de la densité des larves de moustiques

La densité moyenne par louchée (350 ml) de larves de moustiques varie d'un casier rizicole à un autre pour la même pratique agricole. Elle varie également d'une pratique agricole à l'autre et suivant le stade de développement des cultures (tableaux I et II).

**Tableau I:** Densité larvaire moyenne/louchée (350 ml) des populations de moustiques dans les casiers rizicoles selon les pratiques agricoles Average density/dip (350 ml) of mosquito larvae in the rice plots according to agricultural practices

	Repiquage				Tallage				Maturation			
	LP+IP	LP+Iin	LM+IP	LM+Iin	LP+IP	LP+Iin	LM+IP	LM+Iin	LP+IP	LP+Iin	LM+IP	LM+Iin
**Casier 1**	2,25 ±	1,25 ±	1,05 ±	0,3 ±	3,25 ±	1,83 ±	1,05 ±	0,85 ±	0,95 ±	0,5 ±	0,45 ±	0,2 ±
	1,21	0,85	0,94	0,47	1,11	0,93	0,88	0,98	0,88	0,68	0,68	0,41
**Casier 2**	2,45 ±	1,45 ±	0,9 ±	0,25 ±	3,4 ±	2 ±	1,45 ±	0,4 ±	0,55 ±	0,5 ±	0,55 ±	0,2 ±
	1,23	1,23	0,85	0,44	1,09	1,21	1,45	0,50	0,60	0,88	0,75	0,41
**Casier 3**	2,25 ±	1,35 ±	0,9 ±	0,15 ±	3 ±	1,95 ±	2,05 ±	0,3 ±	0,75 ±	0,35 ±	0,55 ±	0,15 ±
	1,37	1,03	0,91	0,36	1,29	0,99	0,94	0,47	0,78	0,67	0,68	0,36

**Tableau II:** Densité moyenne/louchée (350 ml) des larves d'anophèles dans les casiers selon les pratiques agricoles Average density/dip (350 ml) of anopheles larvae in the rice plots according to agricultural practices

	Repiquage				Tallage				Maturation			
	LP+IP	LP+Iin	LM+IP	LM+Iin	LP+IP	LP+Iin	LM+IP	LM+Iin	LP+IP	LP+Iin	LM+IP	LM+Iin
Casier 1	1,15 ±	0,15 ±	0,7 ±	0,25 ±	1,4 ±	0,25 ±	1 ±	0,3 ±	0,35 ±	0,1 ±	0,4 ±	0,05 ±
	0,98	0,36	0,8	0,55	1,53	0,55	1,07	0,47	0,67	0,3	0,68	0,22
Casier 2	1,75 ±	0,2 ±	0,55 ±	0,25 ±	1,4 ±	0,35 ±	0,85 ±	0,25 ±	0,25 ±	0	0,15 ±	0,1 ±
	1,11	0,52	0,68	0,55	1,78	0,58	0,93	0,55	0,71		0,36	0,3
Casier 3	2,15 ±	0,1 ±	0,95 ±	0,05 ±	1,05 ±	0,4 ±	1,15 ±	0,3 ±	0,35 ±	0,1 ±	0,1 ±	0,05 ±
	2,30	0,30	1,05	0,22	0,94	0,75	1,03	0,57	0,81	0,3	0,44	0,22

Le labour minimal combiné à l'irrigation intermittente (LM + Iin) a réduit significati-vement la densité des larves de moustiques, toutes espèces confondues (p = 0,0001) (Tableau III). Les taux de réduction ont été respectivement de 10,5; 5,4 et 2,5 pendant le repiquage, le tallage et la maturation. En considérant seulement les larves d'anophèles, LM + Iin a permis d'obtenir des taux de réduction de la densité larvaire de 16; 5,5 et 4 respectivement pendant le repiquage (p = 0,0001), le tallage (p = 0,0001) et la maturation (p = 0,0089) (Tableau IV).

**Tableau III:** Production de larves de moustiques (pour 350 ml) selon les stades de développement des cultures et selon les techniques culturales Mosquito larval density according to crop development stages and cultural techniques

		Techniques de culture
			LP+IP	LP+Iin	LP+IP	LP+Iin	LP+IP	LP+Iin
**Repiquage**	**Densité larvaire**	2,31	1,35	2,31	0,95	2,31	0,23
	**Taux de réduction**	0,7		1,56		10,5	
	**P value**	< 0,0001		< 0,0001		< 0,0001	
**Tallage**	**Densité larvaire**	3,23	1,93	3,23	1,52	3,23	0,51
	**Taux de réduction**	0,68		1,13		5,4	
	**P value**	< 0,0001		< 0,0001		< 0,0001	
**Maturation**	**Densité larvaire**	0,75	0,45	0,75	0,52	0,75	0,18
	**Taux de réduction**	0,75		0,4		2,5	
	**P value**	0,0206		0,0704		< 0,0001	

**Tableau IV:** Production de larves d'anophèles (pour 350 ml) selon les stades de développement des cultures et selon les techniques culturales Anopheles larval density according to crop development stages and cultural techniques

		Techniques de culture					
		LP+IP	LP+Iin	LP+IP	LP+Iin	LP+IP	LP+Iin
**Repiquage**	**Densité larvaire**	1,68	0,15	1,68	0,73	1,68	0,18
	**Taux de réduction**	16		1,41		16	
	**P value**	< 0,0001		0,0001		< 0,0001	
**Tallage**	**Densité larvaire**	1,28	0,33	1,28	1	1,28	0,28
	**Taux de réduction**	3,33		0,3		5,5	
	**P value**	< 0,0001		0,2046		< 0,0001	
**Maturation**	**Densité larvaire**	0,32	0,06	0,32	0,22	0,32	0,06
	**Taux de réduction**	4		0,5		4	
		**P value**	0,0089		0,3697		0,0089	

### Impact des technologies expérimentées sur le rendement du riz

Les rendements moyens des différents essais ne sont pas significativement différents d'une technique culturale à l'autre au seuil de 5% (F = 1,07; ddl1 = 3; ddl2 = 11; p = 0,415) (Tableau V).

**Tableau V:** Rendement en kg/superficie de casier (16,5 m^2^) de la Yield of production in kg/field area (16.5 m^2^) according to production suivant les essais assay

Casiers rizicoles	**Rendement (kg/16,5 m2)**			
	LP+IP	LP+Iin	LM+IP	LM+Iin
**Casier 1**	6,93	5,94	5,94	5,28
**Casier 2**	5,20	4,62	5,78	5,78
**Casier 3**	6,19	5,61	5,28	5,28
**Quantité moyenne ± écart type**	6,10 ±0,87	5,39 ±0,69	5,66 ±0,34	5,44 ±0,28

## Discussion

La présente étude a montré que l'irrigation permanente était la seule méthode d'arrosage des casiers rizicoles utilisée par les producteurs de Malanville. Cette méthode d'irrigation a déjà été utilisée par des producteurs sur d'autres périmètres rizicoles en Afrique, notamment au Burkina Faso dans la vallée du Kou [18], en Côte d'Ivoire [15] et en Éthiopie [4]. Le labour profond était également la seule méthode de labour pratiquée par les agriculteurs. L'agriculture irriguée est nécessaire à l'expansion de la productivité agricole pour assurer la sécurité alimentaire [12]. Cependant, l'irrigation permanente des casiers génère des lieux de ponte favorables aux moustiques du genre *Anopheles* qui sont responsables de nuisances et de la transmission du paludisme. La présence permanente d'eau dans les casiers rizicoles constitue un facteur déterminant de la prolifération des anophèles et de la transmission du paludisme dans la communauté des producteurs de Malanville [1].

Dans cette étude, de nouvelles pratiques agricoles ont été évaluées quant à la réduction de la production de larves de moustiques dont les anophèles. Ces nouvelles pratiques étaient l'irrigation intermittente associée au labour minimal. Les essais réalisés ont montré que l'irrigation intermittente réduit significativement la densité de moustiques et des anophèles lorsque le riz est cultivé sur du labour minimal (profondeur d'environ 5 cm) par rapport aux casiers à irrigation permanente et au labour profond (profondeur 15-25 cm). Le niveau de réduction était variable suivant le cycle de développement, du repiquage à la maturation en passant par le tallage. Des résultats similaires ont été rapportés en Éthiopie, où la densité des anophèles dans les casiers rizicoles à irrigation permanente était 3,6 fois plus élevée que celle enregistrée sur des parcelles à irrigation intermittente [13]. En outre, des études menées sur des parcelles rizicoles de Fanaye (Sénégal) ont montré que grâce à l'alternance de l'arrosage et de l'assèchement, le volume d'eau d'irrigation peut être réduit de 20 à 50% sans diminuer le rendement de la production du riz [5].

À la fin des essais, les quantités de riz récoltées n'étaient pas significativement différentes entre les casiers à irrigation permanente et ceux à irrigation intermittente. Ces résultats confirment ceux de Djaman *et al.* [5]. Toutefois, en tenant compte de la petitesse des superficies exploitées pour les essais, il serait prématuré de conclure sur le rendement des récoltes. Ces essais nécessitent d'être repris sur de plus grandes superficies afin de préciser les résultats.

Les producteurs sont convaincus de l'apport positif du système de labour minimal et d'irrigation intermittente du point de vue de la balance de rentabilité économique et du gain sanitaire probable. Cependant, les contraintes relatives au nombre d'années d'expérience dans la production du riz, à l'appartenance du producteur à un groupement, à la disponibilité de la main-d'œuvre ou encore à l'insuffisance de ressources financières sont des facteurs susceptibles de freiner l'adoption du système de labour minimal et d'irrigation intermittente. Des séances d'information et de sensibilisation de routine devront être organisées afin de convaincre les agriculteurs d'un changement de comportement en ce qui concerne la gestion de l'eau et des sols dans les agrosystèmes irrigués.

Le système de labour minimal et d'irrigation intermittente représente une bonne option pour la lutte contre les vecteurs du paludisme dans les rizières. Il doit être modulable et adapté aux conditions locales des paysans. La réduction effective de l'incidence palustre par l'irrigation intermittente et le labour minimal ne peut être évaluée ou quantifiée à travers la présente étude. Une étude comparative à plus grande échelle incluant des rizicultures à irrigation permanente et à irrigation intermittente dans la même zone agroécologique serait nécessaire afin d'évaluer la réduction de l'incidence des accès palustres liée à l'irrigation intermittente.

## Conclusion

Le système d'irrigation intermittente associée au labour minimal paraît performant dans la réduction de la densité des larves de moustiques dont celles des anophèles dans les rizières. L'adoption de cette nouvelle technologie contribuerait à réduire l'incidence du paludisme chez les populations riveraines. Cependant, il est souhaitable de mener des études complémentaires afin de prouver aux riziculteurs l'effet de l'irrigation intermittente sur le rendement et sur le gain sanitaire.

## Remerciements

Nous présentons nos chaleureux remerciements à la communauté de Malanville et plus particulièrement aux riziculteurs pour leur coopération et leur assistance durant les travaux de terrain.

## Contribution Des Auteurs

ID, MA, RD ont conçu le protocole de l'étude. ID, YLEL, DHD ont collecté les données, analysé et interprété les données statistiques. ID, YLEL, MKYGB, DHD ont rédigé le manuscrit avec la collaboration de l'ensemble des auteurs. BG, RA, RD, FC ont apporté leur expertise dans la conception de l'étude et ont fait des apports significatifs dans la finalisation du manuscrit. Tous les auteurs ont lu et approuvé le manuscrit final à soumettre pour publication.

## Liens d'intÉrêts

Les auteurs ne déclarent aucun conflit d'intérêts.
